# Equal Opportunities at the Net: Geometric Insights Into Volleyball Scaling

**DOI:** 10.1002/ejsc.70184

**Published:** 2026-05-26

**Authors:** Ole M. Kleivenes, Per K. Rekdal

**Affiliations:** ^1^ Faculty of Business Administration and Social Sciences Molde University College Molde Norway; ^2^ Faculty of Logistics Molde University College Molde Norway

**Keywords:** geometric scaling, net height, sex‐based differences, spike reach, volleyball

## Abstract

This paper investigates how volleyball net height can be scaled to account for differences in vertical spike reach. Although such differences are not exclusively determined by sex, the observed gap between male and female players makes sex‐based comparisons particularly relevant. The official net heights of 243 cm for men and 224 cm for women differ by 19 cm, a regulation intended to compensate for physical disparities between the sexes. We examine whether this adjustment ensures comparable offensive opportunities when evaluated in terms of observed spike reach. To isolate the geometric effect, other performance‐related factors are deliberately disregarded. Using a geometric model linking net height to spike reach, our analysis shows that the current 19 cm gap is insufficient to ensure equal attacking opportunities. At the elite level, geometric equivalence would require lowering the women's net to about 213 cm or raising the men's net to about 253 cm. The disadvantage faced by female players becomes even more pronounced at lower performance levels.

## Highlights

- A geometric scaling model is developed to determine volleyball net heights that provide comparable offensive opportunities for male and female players based on their vertical spike reach.
- The current 19 cm gap between the men's net height of 243 cm and the women's net height of 224 cm is insufficient to ensure equal attacking conditions when evaluated in terms of observed spike reach.
- At the elite level, geometric equivalence would require lowering the women's net to about 213 cm or raising the men's net to about 253 cm, corresponding to corrections of 11 cm and 10 cm, respectively.
- The disadvantage faced by female players under the current scaling becomes even more pronounced at lower performance levels, where the mismatch grows with decreasing spike reach.

## 1 Introduction

Scaling has been applied in many sports to account for anthropometric and physiological differences between men and women, with the aim of enabling them to perform their sport in a comparable manner. As argued by Pedersen et al. (2019), a lack of appropriate scaling may contribute to an underappreciation of female performance.

Although women's volleyball is widely popular, and in some countries even more so than men's (FIVB 2024; Glasspiegel 2025; Konopka et al. 2025), we nonetheless argue that the point made by Pedersen et al. remains important. To fully recognize female performance, it is valuable to understand how the differentiation of net height affects male and female execution of the sport.

Net height is a critical factor influencing gameplay dynamics, particularly in the attack phase, and thus represents the central mechanism through which volleyball seeks to provide equal conditions for both sexes. In volleyball, scaling is implemented through different net heights, set to 243 cm for men and 224 cm for women at the senior level.

Despite the current scaling effort, several dissimilarities remain in how men and women play the game. Differences have been documented both in the efficacy of various phases of play (Kountouris et al. 2015; Ciemiński 2018; Palao et al. 2009) and in tactical choices (Lima et al. 2019). In addition, kinematic analyses have shown that female players' jumping and hitting technique appears less optimal than that of male players (Slovák et al. 2024; Slovák et al. 2026; Fuchs et al. 2019). Such technical differences may, however, reflect coordinative strategies adapted to the sex‐specific constraints faced by the players (Fuchs et al. 2019). This would be in line with the constraints‐led approach to motor learning (Newell 1986; Davids et al. 2008), in that if the scaling of the net height does not sufficiently account for anthropometric and physiological sex differences, women might adopt technical solutions that appear sub‐optimal when evaluated against male performance, but necessary due to the task constraints they are facing.

Taken together, these observations make it reasonable to question whether the current net height regulation provides equal conditions for both sexes. We have not been able to find any official explanation for how the current scaling of the net was determined. Still, we note that the 19 cm difference between the men's and women's net heights is not far from the average sex‐based difference in overhead fingertip reach of 16.99 cm reported by Gordon et al. (1989, 129).

One could, however, argue that, since most plays at the net are performed while jumping, a proper scaling calculation should be based on differences in maximum spike reach rather than standing reach. Using data from the Olympics and World Championships between 2000 and 2012, Palao et al. (2014, 230) found in their study average reach values of 343 cm for male and 301 cm for female players, respectively. This gives a 42 cm difference between the sexes. By comparison, the net is only 19 cm higher for men than for women, giving men a seemingly considerable advantage when attacking.

The smaller the gap between spike reach and net height, the narrower the timing window for spiking the ball, increasing the player's risk of error in offensive play. This also affects spiking angles and the size of the targetable court area. To counter this inequality between sexes, extending the net height difference to 42 cm may seem a straightforward solution. However, although this adjustment addresses the timing issue, it does not fully resolve the matter of angles and targetable area, which depend not only on the spike reach/net height ratio but also on the absolute net height.

The issue of fair scaling of net height in volleyball is by no means straightforward (see, e.g., Palao et al. 2024). Among the various factors that shape equal playing conditions between men and women, the geometric relationship between spike reach and net height deserves careful consideration, particularly in the attack phase of the game. In this paper, we derive a simple mathematical model to explain how net height could be adjusted to account for differences in spike reach, with the aim of creating comparable opportunities in terms of targetable area and spiking angles. We are aware, however, as shown for example by Palao et al. (2024), that altering the net height affects gameplay dynamics in various ways, some of which may directly or indirectly influence players' attacking efficacy. We emphasize that it is beyond the scope of this paper to account for all such factors. The intention of this paper is not to establish the ideal scaling of net height in volleyball, but to provide a starting point for further discussions.

## 2 Model and Scaling Formula

**FIGURE 1:**
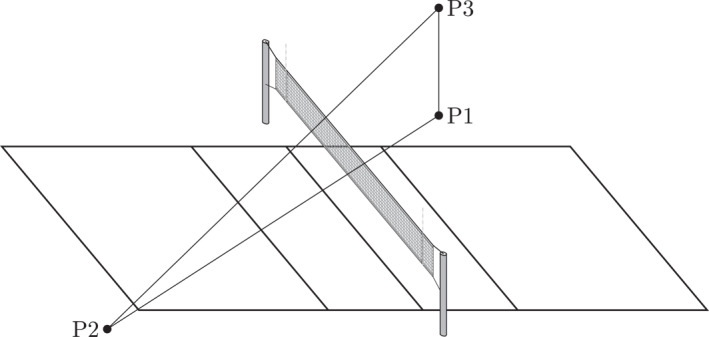
Geometric illustration of the model under consideration. The player takes off from point P1, performs the spike at P3, and the ball then travels in a straight line to land at point P2 on the opposite side of the net. The male and female players share the same ground location P1 and target the same landing point P2, whereas the contact point with the ball occurs at maximum reach height P3. The specific locations of P1 and P2 are general and may lie anywhere on either side of the net, whether inside or outside the court boundaries.

Throughout this paper, we consider two groups of players with different spike reach. For concreteness, we refer to them as male and female players. We assume that both groups perform a vertical jump from the same ground location on one side of the net, denoted by P1, and smash the ball toward a common landing point on the opposite side, denoted by P2, as illustrated in Figure 1. Although the point of contact between the ball and the player's hand, denoted by P3, may vary due to differences in spike reach, the ground‐to‐ground trajectory (P1‐P2) remains the same. This geometric assumption provides a consistent basis for comparing trajectories and deriving analytical relationships between spike reach and net height for the two groups.

As also illustrated in Figure 1, we emphasize that the positions P1 and P2 are not restricted to locations within the court boundaries. The analysis remains valid regardless of whether the starting position P1 and/or target position P2 lie inside or outside the court. It holds irrespective of whether the smash is, for example, directed diagonally, straight down the sideline, or along any other trajectory across the net.

In addition, several further idealizations are introduced to allow for a tractable geometric analysis. The point of contact between the player and the ball (P3 in Figure 1) is assumed to coincide with the vertical spike reach, and the ball itself is modeled as a point object, that is, no spatial extent. Its trajectory is idealized as a straight line, neglecting effects such as, for example, spin, air resistance, and gravity. The ball is further assumed to pass tangentially just above the net, without making contact, that is, its path is not influenced by any interaction with the net. Likewise, the landing point (P2 in Figure 1) on the opponent's side is also treated as a point. This idealized setup enables a simplified geometric treatment of the relationship between spike reach and net height for the two groups considered. Table 1 summarizes these assumptions and their implications.

**TABLE 1:** Summary of model assumptions and what the model does not account for.

The model assumes	The model does not account for
Ball is a point object	Ball size and spin
Straight‐line trajectory	Gravity, air resistance, curved paths
Ball passes tangentially above the net	Net contact or deflection
Vertical jump	Horizontal movement during takeoff
Spike at maximum vertical reach	Variation in contact height or timing
Same takeoff point and landing point for both groups	Differences in horizontal positioning
Takeoff and landing points are general	Court boundaries as constraints
Two groups differing only in spike reach	Other physiological or tactical differences

Under the assumptions described above, there emerges a direct and analytically tractable relationship between spike reach and net height for male and female players. Let $r_{m}$ denote the vertical spike reach for male players, and $r_{f}$ the corresponding spike reach for female players. Also, let $h_{m}$ and $h_{f}$ be the net heights for male and female players, respectively. An overview of the key variables and parameters used throughout the paper is given in Table 2. These quantities are then related by the scaling formula:

(1) $$\frac{h_{f}}{h_{m}}=\frac{r_{f}}{r_{m}},$$

as shown in the Appendix.

**TABLE 2:** Overview of key variables and parameters used in the scaling model.

Symbol	Description	Definition
$r_{m}$	Vertical spike reach for male players	Figure A1
$r_{f}$	Vertical spike reach for female players	Figure A1
$h_{m}$	Net height for male players	Figure A1
$h_{f}$	Net height for female players	Figure A1
R	Difference in spike reach between sexes	$R=r_{m}-r_{f}$
H	Difference in net height between sexes	$H=h_{m}-h_{f}$
ε	Parameter representing variation in spike reach	Sections 3.1.1 and 3.1.2

## 3 Net Heights

We now explore the implications of the scaling formula in Equation (1), examining how volleyball net height should be adjusted due to differences in spike reach. We analyze two specific cases: first, assuming a fixed reach difference between the two groups, and second, allowing this difference to vary.

### 3.1 Fixed Difference in Reach Heights

As a first step toward understanding the scaling formula, we consider the case where the spike reach difference between male and female players is assumed constant. As noted in the introduction, elite‐level data suggest a gap of approximately 40 cm, which we now assume fixed across the entire range of spike reach.

#### 3.1.1 Male Net Height

Let us now consider a fixed female net height, for example,

(2) $$h_{f}=224\;\text{cm},$$

which is the official net height for women in international competition. In line with our assumption, we take male players to have a spike reach of 40 cm higher than females. We also require $r_{f}>h_{f}=224\;\text{cm}$, as dictated by the geometric constraint discussed in the Appendix A. Based on this, we define spike reach as follows.

(3) $$r_{m}=264+\varepsilon \;\text{cm},$$

(4) $$\begin{array}{ll} r_{f} & =224+\varepsilon \;\text{cm}, \end{array}$$

where ε>0 is a parameter representing variation in spike reach. We can now compute the implied net height for men by substituting these expressions into the general scaling formula in Equation (1), obtaining:

(5) $$h_{m}=\frac{r_{m}}{r_{f}}h_{f}=\frac{264+\varepsilon }{224+\varepsilon }\;\cdot \;224\;\text{cm}.$$

**FIGURE 2:**
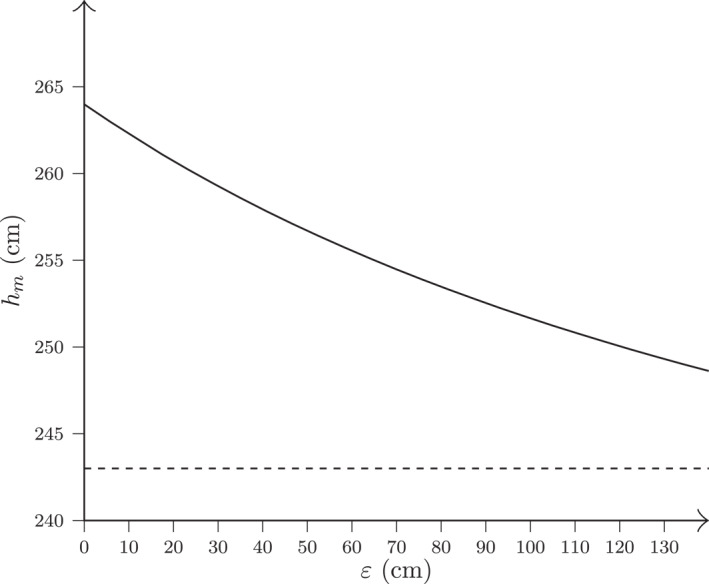
The male net height $h_{m}$ as a function of ε according to Equation (5) for a fixed female net height $h_{f}=224$ cm and fixed spike reach difference between the sexes at $r_{m}-r_{f}=40$ cm. The dashed horizontal line represents the official male net height of 243 cm, included for reference.

By varying ε within 0<ε<130 cm, we span a broad range of realistic spike reach values (corresponding to $264<r_{m}<394$ cm and $224<r_{f}<354$ cm), covering most levels of competitive play. As illustrated in Figure 2, the resulting male net height remains above 243 cm for all values of ε>0, indicating that the model suggests a higher net height for men than the current official height.

As an example of the implications of Equation (5), we consider ε=80 cm, corresponding to a male spike reach of $r_{m}=264+80=344$ cm and female spike reach of $r_{f}=224+80=304$ cm, values typical of elite‐level play. Equation (5) then yields:

(6) $$h_{m}=\frac{344}{304}\;\cdot \;224=253\;\text{cm},$$

where the value is rounded to the nearest centimeter. This means that our model, when assuming a fixed reach difference of 40 cm, implies that men would require a net height of 253 cm instead of the current 243 cm to match the conditions set by the women's net height of 224 cm. This result demonstrates an under‐scaling of 10 cm, which leads to systematically different conditions for executing smashes between sexes.

#### 3.1.2 Female Net Height

We now turn to the inverse scenario, where the male net height is held fixed and we instead compute the corresponding net height for women under the same geometric assumptions. Following the same approach as in the previous section, we consider a fixed male net height equal to the official value $h_{m}=243$ cm. Again, assuming a fixed reach difference of 40 cm, we can write.

(7) $$r_{m}=243+\varepsilon \;\text{cm},$$

(8) $$r_{f}=203+\varepsilon \;\text{cm}.$$

With ε>0 we also realize that $r_{m}>h_{m}=243$ cm, as required by our model. We then compute the corresponding net height for women. Substituting these expressions into the general scaling formula from Equation (1), we derive the following expression for the female net height:

(9) $$h_{f}=\frac{r_{f}}{r_{m}}h_{m}=\frac{203+\varepsilon }{243+\varepsilon }\;\cdot \;243\;\text{cm}.$$

**FIGURE 3:**
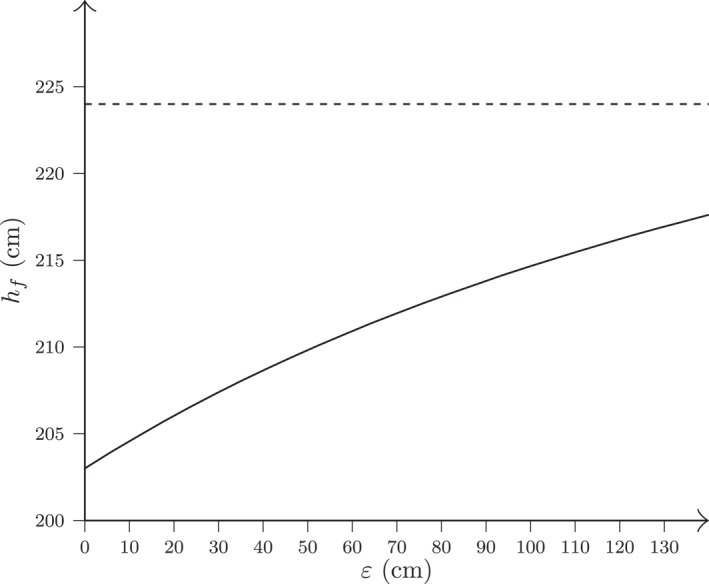
The female net height $h_{f}$ as a function of ε according to Equation (9) for a fixed male net height $h_{m}=243$ cm and fixed spike reach difference between the sexes at $r_{m}-r_{f}=40$ cm. The dashed horizontal line represents the official female net height of 224 cm, included for reference.

Again, by varying ε within 0<ε<130 cm, we span a broad range of realistic spike reach values ($243<r_{m}<373$ cm and $203<r_{f}<333$ cm), covering most levels of competitive play. As illustrated in Figure 3, the resulting female net height remains below 224 cm for all values of ε>0, indicating that the model suggests a lower net height for women than the current official height.

As an example of the implications of Equation (9), let us consider the case ε=80 cm as before, that is, corresponding to a male spike reach of $r_{m}=243+80=323$ cm and female spike reach of $r_{f}=203+80=283$ cm. With $h_{m}=243$ cm, we then obtain

(10) $$h_{f}=\frac{283}{323}\;\cdot \;243=213\;\text{cm},$$

where the value is rounded to the nearest centimeter. That is, according to our model, to preserve equivalent offensive geometry with $h_{m}=243$ cm and a 40 cm reach gap, women should play with a net height of 213 cm, that is, 11 cm lower than the current standard. This reinforces the conclusion that the present net height difference between men and women may be insufficient to produce equivalent geometrical conditions for executing smashes between sexes.

### 3.2 Net Height Difference and Spike Reach Difference

To gain further insight into the scaling formula, we now express it in terms of difference in spike reach and difference in net height between sexes, rather than their absolute values. Specifically, we define.

(11) $$H=h_{m}-h_{f},$$

(12) $$\begin{array}{ll} R & =r_{m}-r_{f}. \end{array}$$

Here, H denotes the difference in net height between men and women, whereas R represents the corresponding difference in spike reach. Substituting these definitions into the scaling formula in Equation (1) yields:

(13) $$H=\frac{h_{m}}{r_{m}}R.$$

This equation predicts a linear relationship between H and R, where the proportionality constant depends on the ratio of the net height and the spike reach. It is also worth noting that the scaling relation in Equation (13) does not favor either sex. That is, one could equivalently express it as $H=\frac{h_{f}}{r_{f}}R$, yielding the same result due to Equation (1). This symmetry implies that the model treats both sexes on equal footing, that is, the same scaling relation applies regardless of whether male or female parameters are used.

To illustrate the implications of Equation (13), we consider two representative endpoints. For high spike reach values, we now apply $r_{m}=390$ cm, reflecting the upper end of observed values among elite male players. Although this choice is not unique, modest changes in the assumed elite reach would not significantly affect the conclusions drawn from the model. With the official men's net height of $h_{m}=243$ cm, we then obtain

(14) $$H=\frac{243}{390}R=0.6231R,$$

where the proportionality factor is given with four decimal accuracy.

For lower spike reach values, we use $r_{m}=290$ cm for male players. Moreover, in order for players to execute successful smashes from behind the attack line (e.g., from the 300‐cm zone), a reach substantially exceeding the net height is typically required. The value of 290 cm thus represents a plausible and functionally relevant lower bound for male players. As for the lower end of the spike reach, this choice is not unique and moderate adjustments would not significantly alter the model's implications. We now obtain

(15) $$H=\frac{243}{290}R=0.8379R,$$

where the proportionality factor is again reported to four decimal places.

**FIGURE 4:**
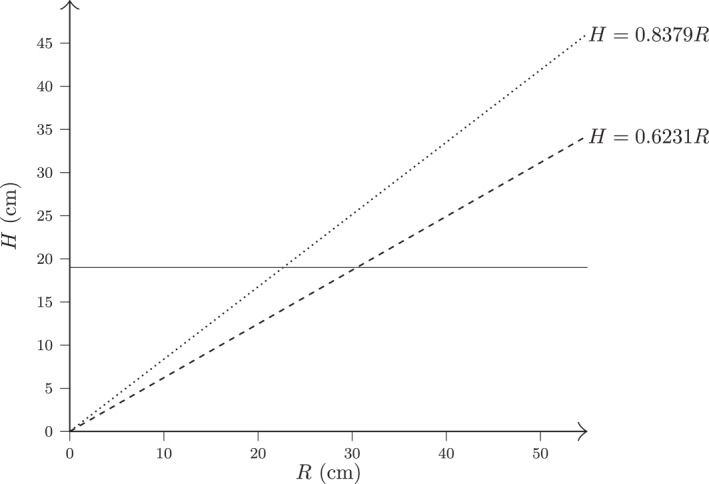
Computed net height difference H as a function of spike reach difference R. The dashed line shows Equation (14) and corresponds to high level of spike reach. The dotted line shows Equation (15) and corresponds to lower level of spike reach. We note that a lower level of spike reach should, according to the scaling formula, correspond to higher net height difference.

Equations (14) and (15) are illustrated graphically in Figure 4. For comparison, both graphs in this figure assume, as mentioned above, the official men's net height of $h_{m}=243$ cm. The dashed line, based on a male spike reach of 390 cm, reflects a high‐reach scenario and yields a proportionality constant of 0.6231. The dotted line, based on a lower spike reach of 290 cm, results in a larger slope 0.8379. In other words, for a given net height, the model suggests that the lower the spike reach, the greater the required height difference must be to preserve equal spiking conditions.

As specific cases from Figure 4, we consider two distinct values of the spike reach difference R. First, for R=40 cm, Equation (14) yields H=25 cm, whereas Equation (15) gives H=34 cm. Both values exceed the current official net height difference of 19 cm (243 – 224 cm) between men and women. Second, for R=30 cm, Equation (14) gives H=19 cm, matching the official difference, whereas Equation (15) produces a larger value of H=25 cm. These examples highlight that, particularly at lower levels of spike reach, the current net height gap may be insufficient to support equivalent attacking opportunities across performance levels.

## 4 Discussion

The aim of this study has been to show how the net height in volleyball can be adjusted to account for the sex‐based differences in spike reach. For practical reasons, we used spike reach data from top international tournaments (Palao et al. 2014) to provide a well‐defined reference level for the comparison. Data from elite‐level tournaments indicate a sex‐based difference in spike reach of approximately 40 cm, and we assume that this difference remains constant irrespective of what level the comparison is made on. We are, however, aware that both anthropometric and physiological differences between boys and girls become larger during puberty, so we understand our assumption is probably only valid for post‐pubertal age groups (Kirchengast 2010).

The current regulations do not reflect the combined anthropometric and physiological differences between men and women, giving women a clear disadvantage in the attack phase of the game, both when it comes to spiking options and timing. We stress that we have only looked at equality between sexes regarding spiking angles and target area, including both in‐court as well as out‐of‐court landing zones of the ball. As pointed out, the smaller the gap between spike reach and net height, the narrower the timing window for spiking the ball becomes, increasing the risk of player error. However, our model is based on an ideal setting, where players always spike at their highest point, thereby eliminating any practical differences in timing window. With that said, we acknowledge that players in the real world do not always hit the ball at the top of their trajectory.

The core insight of this study is that our geometric model confirms substantially more challenging conditions for women than for men in the attacking phase of volleyball due to net heights being insufficiently scaled compared to differences in spike reach. In addition, the degree of inequality depends on the spike reach values being compared. When players with lower reach level are compared, the disparity is greater than when players with higher reach level are considered. This means that deciding a net height that would be fair to both sexes is not straightforward, but requires knowledge about men's and women's spike reach at different playing levels, which would be much more difficult to determine.

Although we have access to average spike reach at the highest level, data for the lowest levels are lacking. We therefore base our analysis in Section 3.1.1 on the women's international net height of $h_{f}=224$ cm with the lowest meaningful reach defined as any value exceeding this height, that is, $r_{f}>h_{f}$. Using this as a baseline, we vary the parameter ϵ over a sufficiently wide interval to span the range of competitive play. Thus, regardless of whether we consider high or low reach levels within this framework, the corresponding male net height predicted by the model lies substantially above the current official value of 243 cm. An analogous result would follow if the analysis were inverted and based on the men's net height instead of the women's, as shown in Section 3.1.2.

To even out the current inequalities, one can either raise the net for men, lower it for women, or apply a combination of both. What to choose depends on whether it is preferable to diminish the spiking angles/target area for men, or to increase it for women. A consequence of the first option might be that male players on the lower end of the spike reach scale, would struggle to perform efficient attacks, which could reduce their enjoyment of the sport. Lowering the women's net height will not affect the men but make all female players more efficient attackers. Following this line of thought, and in accordance with our geometric model, lowering the women's net might seem to be a sensible way forward if one should consider making any changes. However, altering the net height could have implications for other parts of the game, and any adjustments to the current net regulations should involve a broader consideration than we have done in this paper. It might be that the current difference between men's and women's net height is optimal for the total playing dynamic. Moreover, changes are not necessarily welcome in themselves, as illustrated by the debate in basketball on lowering the rim for women (Cash 2021).

A natural next step in the discussion of scaling in volleyball is to include court size. This is of interest in its own right from a scaling standpoint; furthermore, as demonstrated by Palao et al. (Palao et al. 2024), an adjustment of the net may also necessitate a corresponding adjustment of court size. In ongoing work, one of the authors and colleagues are developing a more holistic analysis that incorporates court size together with a broader set of anthropometric and physiological variables (Kleivenes et al. 2026).

The complex and interdependent dynamics of the game make the consequences of regulatory changes difficult to predict. Reducing the net height would be expected to benefit offensive actions while having an adverse effect on defensive ones, though the magnitude of such effects remains unclear. This highlights the need for experimental studies, which would also provide an opportunity to investigate players' subjective experiences. For instance, is it more satisfying to be successful in attack or in defense? If proposed changes are not well received in terms of how engaging the game is to play, they may not be worthwhile.

Another finding worth exploring further is that the optimal net height difference predicted by our model increases at lower levels of spike reach. Since players generally jump considerably lower in sand than on a hard surface, a separate analysis of beach volleyball would be a valuable extension. Such an analysis would, however, present additional challenges compared with indoor volleyball. Although spike reach data for top‐tier indoor players are available from official sources, comparable data for beach volleyball are scarce. In addition sand conditions may vary between courts, making jump‐height comparisons more difficult. A beach volleyball analysis along the lines of the present study would therefore require additional work to identify spike reach differences between men and women under conditions that are both standardised and representative of competitive play.

Given the many perspectives and unknown factors regarding potential changes to net height regulations in volleyball, we cannot predict the practical consequences of this paper. Nevertheless, we believe our findings provide valuable insights that may help coaches, players, and spectators better understand and appreciate the performance of both sexes in the attacking phase of the game.

## 5 Conclusion

In this study, we examined an idealized geometric model considering only net height and spike reach, deliberately disregarding all other influencing factors. The model compares two groups of players with different spike reach, such as male and female players, but the framework is more general and can also be applied when evaluating net height regulations for players in, for example, different age groups.

Our analysis shows that the current 19 cm net height difference between men and women does not reflect the roughly 40 cm gap in spike reach observed at the elite level. According to the model, this mismatch puts female players at a disadvantage by narrowing their spiking angles and reducing the range of possible landing areas for the ball on the opponent's court.

To achieve geometric equivalence, the model allows for several possible adjustments, such as lowering the women's net to 213 cm or raising the men's to 253 cm. These values should be regarded as illustrative examples rather than prescriptive recommendations. The asymmetry is even more pronounced at lower levels with lower spike reach.

Although simplified, our model highlights a structural asymmetry in current regulations. Given the complexity of the game, we are not able to predict the consequences such a change would have on the attractiveness, popularity, and media attention of women's volleyball. Nevertheless, we hope our findings provide valuable insights that may help coaches, players, and spectators better understand and appreciate the performance of both sexes in the attacking phase of the game.

## Funding

The authors have nothing to report.

## Conflicts of Interest

The authors declare no conflicts of interest.

## Appendix A: Derivation of the Scaling Formula

**FIGURE A1:**
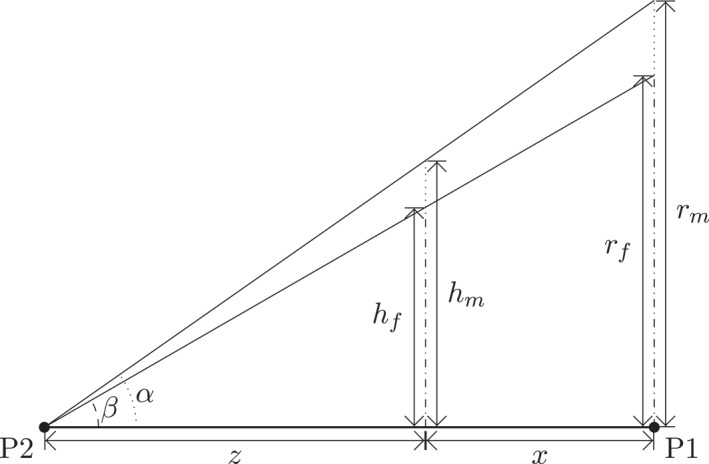
Geometric construction used to derive the scaling formula showing the relation between net height ($h_{m}$ and $h_{f}$) and player reach ($r_{m}$ and $r_{f}$). The figure shows two triangles corresponding to male and female spike trajectories, both originating from the same ground point (P2) and targeting the same impact point (P1). The triangles differ only in vertical extent, reflecting differences in reach and net height.

To derive the relationship between net height and spike reach under the assumptions described in Section 2, we consider the two right‐angled triangles illustrated in Figure A1. The triangles share a common line P1‐P2, and the smash trajectories for male and female players are assumed to form geometrically similar configurations from the same point P1 on the ground, and targeting the same point P2 of impact.

From the triangle formed by the male player's trajectory, we realize that

(A1) $$\frac{h_{m}}{z}=\frac{r_{m}}{z+x}.$$

Here, x denotes the horizontal distance from the net to point P1, whereas z represents the horizontal distance from the net to the landing point P2, see Figure A1. Similarly, the triangle formed by the female player's trajectory is

(A2) $$\frac{h_{f}}{z}=\frac{r_{f}}{z+x}.$$

Dividing the two latter equations eliminates the common terms, leading to Equation (1).

To ensure that a downward trajectory is possible, the spike reach must exceed the net height for both sexes, that is,

(A3) $$r_{m}>h_{m}\;\text{and}\;r_{f}>h_{f}.$$

This fundamental geometric constraint ensures that a straight‐line trajectory aims downward toward the target point P2.

## Data Availability

Data sharing not applicable to this article as no datasets were generated or analyzed during the current study.
